# Systems biology reveals anatabine to be an NRF2 activator

**DOI:** 10.3389/fphar.2022.1011184

**Published:** 2022-11-16

**Authors:** Dimitris E. Messinis, Carine Poussin, Diogo A. R. S. Latino, Yvan Eb-Levadoux, Remi Dulize, Dariusz Peric, Emmanuel Guedj, Bjoern Titz, Nikolai V. Ivanov, Manuel C. Peitsch, Julia Hoeng

**Affiliations:** PMI R&D, Philip Morris Products S.A, Neuchâtel, Switzerland

**Keywords:** anatabine, Nrf2 activator, Nrf2 activation, Nrf2 pathway, Nrf2, HMOX1, alkaloid, p38 MAPK

## Abstract

Anatabine, an alkaloid present in plants of the *So* *lanaceae* family (including tobacco and eggplant), has been shown to ameliorate chronic inflammatory conditions in mouse models, such as Alzheimer’s disease, Hashimoto’s thyroiditis, multiple sclerosis, and intestinal inflammation. However, the mechanisms of action of anatabine remain unclear. To understand the impact of anatabine on cellular systems and identify the molecular pathways that are perturbed, we designed a study to examine the concentration-dependent effects of anatabine on various cell types by using a systems pharmacology approach. The resulting dataset, consisting of measurements of various omics data types at different time points, was analyzed by using multiple computational techniques. To identify concentration-dependent activated pathways, we performed linear modeling followed by gene set enrichment. To predict the functional partners of anatabine and the involved pathways, we harnessed the LINCS L1000 dataset’s wealth of information and implemented integer linear programming on directed graphs, respectively. Finally, we experimentally verified our key computational predictions. Using an appropriate luciferase reporter cell system, we were able to demonstrate that anatabine treatment results in NRF2 (nuclear factor-erythroid factor 2-related factor 2) translocation, and our systematic phosphoproteomic assays showed that anatabine treatment results in activation of MAPK signaling. While there are certain areas to be explored in deciphering the exact anti-inflammatory mechanisms of action of anatabine and other NRF2 activators, we believe that anatabine constitutes an interesting molecule for its therapeutic potential in NRF2-related diseases.

## 1 Introduction

Anatabine is an alkaloid present in plants of the *Solanaceae* family, including green tomatoes, peppers, eggplant, and tobacco, and it exhibits close structural resemblance to nicotine (Nielsen et al., 2013). *In vivo*, anatabine has been shown to ameliorate Alzheimer disease in mice (Paris et al., 2011; Verma et al., 2015) and Hashimoto thyroiditis in mice (Caturegli et al., 2012) and humans (Schmeltz et al., 2014). On the basis of experiments in mice, anatabine has also been suggested to be effective in treatment of multiple sclerosis (Paris et al., 2013b).

As a result, anatabine was marketed from 2011 to 2014 (Anatabloc, Star Scientific Inc.) as a dietary supplement and as an active ingredient in a facial cream with anti-inflammatory properties. During this period, an open-label case series showed that a facial cream containing anatabine could improve the appearance of skin in patients with mild to moderate rosacea (Lanier et al., 2013a), while an internet-based survey study provided evidence that anatabine supplementation could improve chronic joint pain disorders (Lanier et al., 2013b). More recently, anatabine has been shown to ameliorate intestinal inflammation in mice (Ruiz Castro et al., 2020), inhibit acute and chronic inflammation in mice in a dose-dependent manner (Xia et al., 2021), and improve the outcomes of repetitive mild traumatic brain injury in mice upon a 3-month delayed treatment (Morin et al., 2021).

At the molecular level, anatabine inhibits the activity of two transcription factors that are involved in cellular inflammatory response—NF-κB (nuclear factor-kappa B) and STAT3 (signal transducer and activator of transcription 3)—both *in vivo* (Paris et al., 2013a; Paris et al., 2013b) and *in vitro* (Paris et al., 2011; Paris et al., 2013a). In addition, it decreases lipopolysaccharide (LPS)-induced TNFα (tumor necrosis factor α) and IL-6 (interleukin 6) levels in a dose-dependent manner while increasing the levels of the anti-inflammatory cytokine IL-10 *in vivo* (Xia et al., 2021).

While several molecular mechanisms have been proposed and categorized in detail to explain how phytochemicals function as anti-inflammatory agents (Bellik et al., 2012), it remains unclear which of those could pertain to anatabine’s mechanism(s) of action and how the observed molecular perturbations are linked to its anti-inflammatory activity.

Before investigating the effects of anatabine in the context of inflammation, we wanted to understand its impact on cellular systems and identify molecular networks and pathways that are perturbed by anatabine. To this end, we designed a study to examine the concentration-dependent effects of anatabine on various cell types by using a systems pharmacology approach. The investigations were supported by multiomics measurements (transcriptomics, proteomics, and phosphoproteomics) at different time points. To identify concentration-dependent perturbed biological processes/pathways, we performed linear modeling analysis of transcriptomics data followed by gene set enrichment analyses. To predict the functional partners of anatabine and the involved pathways, we harnessed the L1000 wealth of information and implemented integer linear programming on directed graphs, respectively. We then verified some hypotheses using specific cellular assays. Thus, using an appropriate luciferase reporter cell system, we showed that anatabine treatment results in NRF2 translocation. Finally, by leveraging systematic phosphoproteomic assays, we demonstrated that anatabine treatment resulted in activation of MAPK signaling.

## 2 Materials and methods

### 2.1 Cell culture

In this study, we employed four different cell systems: the cell lines HEK-293, SH-SY5Y, and PMA-differentiated THP-1, and primary human epidermal keratinocytes.

The human embryonic kidney cells HEK-293 (Merck, Buchs, Switzerland) were seeded at 60,000 cells per well in a 96-well plate and cultured in Eagle’s minimum essential medium (EMEM; Gibco, Grand Island, NY, United States) supplemented with 10% FBS (Sigma, Burlington, MA, United States), 1% non-essential amino acids (Merck), 1% sodium pyruvate (Gibco), and 1% penicillin/streptomycin (PenStrep; Gibco).

SH-SY5Y is a human bone marrow neuroblastoma cell line that has previously been used to investigate the potential anti-inflammatory effect of anatabine (Paris et al., 2013a). SH-SY5Y cells (CRL-2266, ATCC, Manassas, VA, United States) were seeded at a density of 15,000 cells per well in a 96-well plate and cultured in a medium consisting of 50% EMEM and 50% Ham’s F12 nutrient mix (Gibco), along with 10% FBS, 1% PenStrep, and 1% L-glutamine (Gibco).

The human leukemia-derived monocytic cell line THP-1 is the most widely used *in vitro* model for primary investigations on human macrophages (Lund et al., 2016). THP-1 cells (Merck) were seeded at 100,000 cells per well in a 96-well plate and cultured in RPMI 1640 medium (Gibco) supplemented with 10% FBS and 1% PenStrep and in the presence of 40 ng/ml phorbol 12-myristate 13-acetate [PMA; Thermo Fisher (Kandel) GmbH, Kandel, Germany]. After 48 h, the culture medium was replaced with fresh medium without PMA and allowed to rest for 24 h before treatment, as previously described (Lund et al., 2016).

Primary human epidermal keratinocytes were included as a relevant cell system for skin. The keratinocytes (Ruwag, Bettlach, Switzerland) of healthy, non-smoking, Caucasian donors aged 20–60 years old, were selected from a donor pool. The keratinocytes were seeded at 17,500 cells per well in a 96-well plate and cultured in KGM-Gold Keratinocyte Growth Medium (Ruwag).

All the four cell systems presented above were seeded at a total volume of 190 μL per well to allow for the addition of 10 μL of 20× anatabine, which was added 24 h after seeding, or the addition of non-PMA media in the case of THP-1 cells.

The same anatabine formulation (WuXi Apptec, Shanghai, China) consisting of racemic anatabine free base (hereafter referred to as “anatabine”; MW: 160.22 g/mol) from a single production batch (Batch B) was used across all experiments.

Anatabine concentrations of less than 400 μM were selected to ensure that at least 80% cell viability was observed after 24 h of exposure. Viability was assessed using CellTiterGlo (Promega, Madison, WI, United States) by measuring the ATP content in each treated sample and comparing them with those of relevant controls, which were typically cells treated only with cell culture medium.

### 2.2 Transcriptomics

To generate transcriptomics data, cells were exposed to different concentrations of anatabine (0, 100, 200, 300, and 400 μM) for 6 h, and their transcriptome was analyzed using a microarray-based technique.

In detail, all three cell lines were lysed using RLT buffer (Qiagen, Hilden, Germany), and keratinocytes were lysed using the Qiazol lysis reagent (Qiagen). RNA isolation was performed with the RNeasy Micro Kit (Qiagen) on a Qiacube instrument (Qiagen). RNA was quantified with Nanodrop 1000 (Thermo Fisher Scientific, Waltham, MA, United States). The quality of the total RNA, which was required to have an RNA integrity number greater than 6.0, was assessed using 2100 Bioanalyzer (Agilent Technologies, Santa Clara, CA, United States). Total RNA (50 ng) was processed in the Tecan/Nugen Ovation RNA Amplification system V2 kit (Tecan, Männedorf, Switzerland), according to the manufacturer’s instructions, followed by cDNA fragmentation and labeling using the Encore Biotine module (Tecan). The Human Genome U133 Plus 2.0 microarray was used for hybridization using a Thermo Fisher Oven 645. Furthermore, washing was performed on Affymetrix GeneChip™ Fluidics Stations 450Dx (protocol FS450-0004) and scanning on a ThermoFisher GeneChip™ Scanner 3000 7G.

The Bioconductor affyPLM package in R version 1.64 (Bolstad et al., 2004) was used for quality control checks of all chips. Following quality control procedures, differential gene expression was analyzed using the Bioconductor limma package in R version 3.44.3 (Ritchie et al., 2015). Pairwise comparisons at the gene level, called systems response profiles (SRP), were computed by comparing each concentration-treatment with its respective vehicle control. Genes with a false discovery rate (FDR) (*p*-value adjusted using the Benjamini and Hochberg method) below 0.05 were considered differentially expressed genes (Benjamini and Hochberg, 1995). SRPs including all genes (∼18,000) were further leveraged in downstream *p*-value threshold-free gene set enrichment analysis (GSEA), as described in Section 2.6.

### 2.3 Data-independent acquisition mass spectrometry

Samples for DIA were prepared using the PreOmics iST kit (PreOmics GmbH, Planegg/Martinsried, Germany), according to the manufacturer’s protocol. Briefly, 100 µL of PreOmics lyse buffer was added to the cells, and the mix was incubated at 90°C for 10 min and sonicated for 30 s with a sonifier (Branson, Danbury, CT, United States) at 10% amplitude. Protein concentration was determined using the Pierce 660 nm protein assay (Pierce Biotechnology Inc., Rockford, IL, United States), according to the manufacturer’s protocol. The samples were normalized to 0.5 μg/μL, and 40 µg of each sample was further processed with the PreOmics iST kit with a 3-h long trypsin digestion. Peptides were purified on the cartridge, dried overnight on a vacuum concentrator (Martin Christ, Osterode, Germany), and resuspended in 50 µL of LC Solution (Biognosys AG, Schlieren, Switzerland). iRT reference peptides (1 μL; Biognosys AG) were added to 19 µL of the processed samples, which were analyzed with Easy 1000 nanoLC (Thermo Fisher Scientific) connected online to a Q-Exactive mass spectrometer (Thermo Fisher Scientific). Two microliters of the peptide mixture were separated on a 0.75 × 500 mm, 1.7 μm C18 column (Thermo Fisher Scientific) using solvent A (1% acetonitrile/99% water/0.1 formic acid) over 115 min with a gradient of 5%–35% solvent B (95% acetonitrile/5% water/0.1 formic acid) at 200 nL/min.

Data were acquired on the Q-Exactive system in the DIA mode: MS1 scan at a 140 k resolution was followed by 23 custom MS/MS *m/z* windows at a 35 k resolution, as previously described (Bruderer et al., 2017) with slight modifications. Data were processed with Spectronaut Pulsar (v. 13.8.190930.43655; Biognosys AG) using the DirectDIA feature.

SRPs were computed by comparing samples at each concentration with their respective vehicle control, as described in Section 2.2.

### 2.4 Phosphoproteomics

Cells were exposed to 100, 200, 300, and 400 μM of anatabine for 15 min, 24 min, 6 h, and 24 h. For each time point, the cells were placed on ice, washed with phosphate-buffered saline, and lysed with the addition of lysis buffer (Protavio Ltd, Cambridge, United Kingdom). The obtained lysed samples were frozen at –80°C. Just before the phosphoproteomic measurement, the samples were quickly thawed and centrifuged for 20 min at 2700 *g* to remove cellular debris. The xMAP assays were performed using a custom-made 18-plex phosphoprotein detection kit (Protavio Ltd), according to the kit instructions, and a Luminex FLEXMAP 3D instrument (Luminex, Austin, TX, United States).

Data were acquired and pre-processed as described previously (Michailidou et al., 2015). SRPs were computed as described in Section 2.2.

### 2.5 NRF2 reporter gene assay

An HEK-293-based NRF2/ARE luciferase reporter cell line (Signosis, Santa Clara, CA, United States) was used to evaluate NRF2 activation. The cell line was stably transfected with pTA-ARE-luciferase reporter vector, which contains four repeats of the antioxidant response binding site, a minimal promoter upstream of the firefly luciferase coding region, along with a hygromycin expression vector. Following a 24-h stimulation of the NRF2 reporter cells with various treatment conditions, luminescence was measured using a FLUOstar Omega plate reader (BMG LABTECH, Ortenberg, Germany). Dimethyl fumarate (Alfa Aesar and Sigma) and sulforaphane, two NRF2 activators, were used as positive controls for the assay. SRPs were computed as described in Section 2.2.

### 2.6 Linear model and GSEA

Linear model analysis was used to identify genes whose expression changed linearly with an increase in anatabine concentration (0, 100, 200, 300, and 400 μM). The linear model analysis was conducted using the transcriptomics data acquired either from each cell system separately (gene expression ∼ β_0_ + β_1_*anatabine_concentration + ε) or all cell systems combined (gene expression ∼ β_0_ + β_1_*anatabine_concentration + β_2_*cell_systems + ε) to identify cell system-specific and cell system-independent genes (“core genes”), respectively. Genes with β_1_ coefficient-associated FDR <0.05 were considered to have a linear change in their expression with increasing concentrations of anatabine. The limma package (version 3.38.3) in R (version 3.5.1) was used for the analysis.

Leveraging these results, GSEA was performed to support biological interpretation (Subramanian et al., 2005). Genes were ranked in decreasing order based on their respective β_1_ coefficient-associated t-statistics. The “C2-CP” (Canonical Pathways) and “C3-TFT” (Transcription Factor Targets) MSigDB (version 7.1) gene set collections were used as sources of *a priori* biological knowledge (Liberzon et al., 2015). Gene sets with normalized enrichment score (NES)-associated FDR <0.05 were considered to be significantly enriched.

### 2.7 L1000 LINCS transcriptomic signature comparison

To investigate the mode of action of anatabine, gene expression profiles obtained in response to anatabine were compared with those obtained in response to a plethora of perturbagens corresponding mostly to chemical/drug compounds (Messinis et al., 2021). These profiles are part of the publicly available level 5 LINCS L1000 dataset (GEO accession number GSE92742), consisting of 473,647 differential gene expression signatures (profiles), created using various concentrations of 28,927 unique perturbagens for treating multiple cell systems. Each LINCS L1000 gene expression “signature” corresponds to the measurement of 978 genes. Genes from anatabine expression profiles generated using oligonucleotide microarray were matched with those from the L1000 dataset, resulting in 938 genes in common that were used for further comparison analysis.

Spearman correlation was used to compare L1000 and genes from expression signatures of anatabine-treated systems. The ranking of genes for anatabine expression signature was based on their respective β_1_ coefficient-associated t-statistics computed using a linear model combining all cell systems. Among the L1000 perturbations, there were several cases where the same compound was used in different experimental conditions. In those cases, the comparison with the highest Spearman correlation coefficient was retained. We ran 100 random transcriptomic signatures against all L1000 signatures in parallel, calculated the significance of the hypothesis that anatabine’s Spearman correlation coefficient values are in the same range as those of the random signatures’, and discarded any observation with a *p*-value greater than 0.001.

### 2.8 Chemical similarity fingerprints and descriptors

To compare the chemical similarity among compounds, the compounds were encoded using several chemical similarity fingerprints and descriptors. The chemical similarity was compared using the DataWarrior software (Sander et al., 2015). DataWarrior was used to calculate the chemical similarity descriptors “Fragment Fingerprints” (FragFP), “Pathway Fingerprints” (PathFp), “OrgFunctions,” “Spheres Fingerprints” (SphereFp), “SkeletonSpheres” (SkelSpheres), and “Flexophore.”

FragFP (Durant et al., 2002) is a substructure fragment dictionary-based binary fingerprint similar to MDL Keys (Xue and Bajorath, 2000), which contains 512 predefined structure fragments. The FragFP descriptor contains 1 bit for each fragment in the dictionary. A bit is set to 1 if the corresponding fragment is present in the molecule at least one time.

PathFp (O'Boyle et al., 2011) encodes any linear fragment of up to seven atoms into a hashed binary fingerprint of size 512 bits. All possible “paths” of seven or less atoms in the molecule are encoded. A text string that encodes atomic numbers and bond orders is generated from the path in a normalized way. From this text string, a hash value is generated that is used to set the corresponding bit of the fingerprint to 1.

SphereFp (Bremser, 1978) encodes circular spheres of atoms and bonds into a hashed binary fingerprint of size 512 bits. Fragments of increasing size are generated by including 1–5 n layers of atom neighbors for every atom in the molecule. A canonical representation of these circular fragments is obtained considering their aromaticity but not their stereo configurations. A hash code is then generated, which is used to set the respective bit of the fingerprint.

SkelSpheres (Boss et al., 2017) is similar to SphereFp but also encodes stereochemistry. Additionally, it counts duplicate fragments, encodes heteroatom depleted skeletons, and has twice the resolution leading to fewer hash collisions (bit size: 1024).

OrgFunctions (Sander et al., 2015) encodes the functional groups in the molecule and the steric or electronic features of the neighborhood of the functional groups. It encodes 1024 core functions that overlap in some cases. Molecules that contain the same functional groups are considered similar irrespective of the possible presence of carbon skeletons.

Flexophore (von Korff et al., 2008) encodes 3D-pharmacophore features of the molecules. This descriptor can be used to check if two compounds have a similar protein binding mode. A high Flexophore similarity of two molecules indicates that a significant fraction of conformers of both molecules is similar with regard to shape, size, flexibility, and pharmacophore features. In addition to a 3D-pharmacophore model, the Flexophore descriptor matches entire conformer sets rather than comparing individual conformers.

### 2.9 Three-dimensional pharmacophore model

Three-dimensional pharmacophore models of subsets of the 15 compounds were built using the Molecular Operating Environment (MOE) software (2019.01; Chemical Computing Group ULC, Montreal, Canada). The pharmacophores were built using the “Pharmacophore Elucidation” tool in MOE. First, a complete conformational search of each individual compound was performed, and similar conformers were removed. The compounds of interest were then aligned, and the best alignment was selected for implementation of the most relevant pharmacophore features of the selected compounds. The alignment was performed giving emphasis to aromatic, donor, and acceptor atoms. The pharmacophore features were generated using the default list of possible features and the default radius of each feature. Finally, from this set of suggested features, the “Pharmacophore consensus” tool was used to select the most relevant pharmacophore features.

### 2.10 Network analysis

Transcriptomics data were used to infer a network model representative of mechanisms of action of anatabine. As a first step, transcriptomics data corresponding to the outcomes of the linear model including all anatabine concentrations and combined cell systems were reverse-engineered to predict potential active transcription factors using the DoRothEA algorithm (Garcia-Alonso et al., 2019). The top 10 most actively predicted transcription factors were selected for further network construction. Ten is an arbitrarily selected number, so as to obtain a resulting network that does not have too many nodes and therefore is possible to visualize.

As a second step, the CARNIVAL algorithm (Liu et al., 2019) was used to build a network leading to the 10 predicted transcription factors and starting by a hypothesized perturbation, which was anatabine in our case. CARNIVAL requires a prior knowledge network, on which the experimental data will be fitted. Two publicly prior knowledge networks were used: OmniPath and Reactome Functional Interactions (FI). OmniPath (Turei et al., 2016) contains 164,710 interactions, whereas Reactome FI (Wu et al., 2010) consists of 259,151 interactions. Both networks have been assembled utilizing numerous resources and are updated regularly. We used the latest versions available for both (version 2.0.0 of OmniPath R package, and the 2020 version of Reactome FI).

The CARNIVAL algorithm generates an integer linear programming problem based on the provided prior knowledge network and the predicted transcription factors and then solves it with the solver IBM ILOG CPLEX. The solution includes the predicted network topology along with a value for every node of the network ranging from 0 to 100, which corresponds to the node’s predicted level of activation. The network topology was visualized using Cytoscape version 3.8.2 (Shannon et al., 2003).

## 3 Results

### 3.1 Gene expression responses to anatabine treatment are characterized by the perturbation of processes associated with cellular redox balance

HEK-293 cells, SH-SY5Y cells, PMA-differentiated THP-1 cells, and primary human keratinocytes were treated with various concentrations of anatabine or vehicle control for 6 h. At the end of the treatment, cell lysates were collected and processed to generate transcriptomic profiles.

**FIGURE 1:**
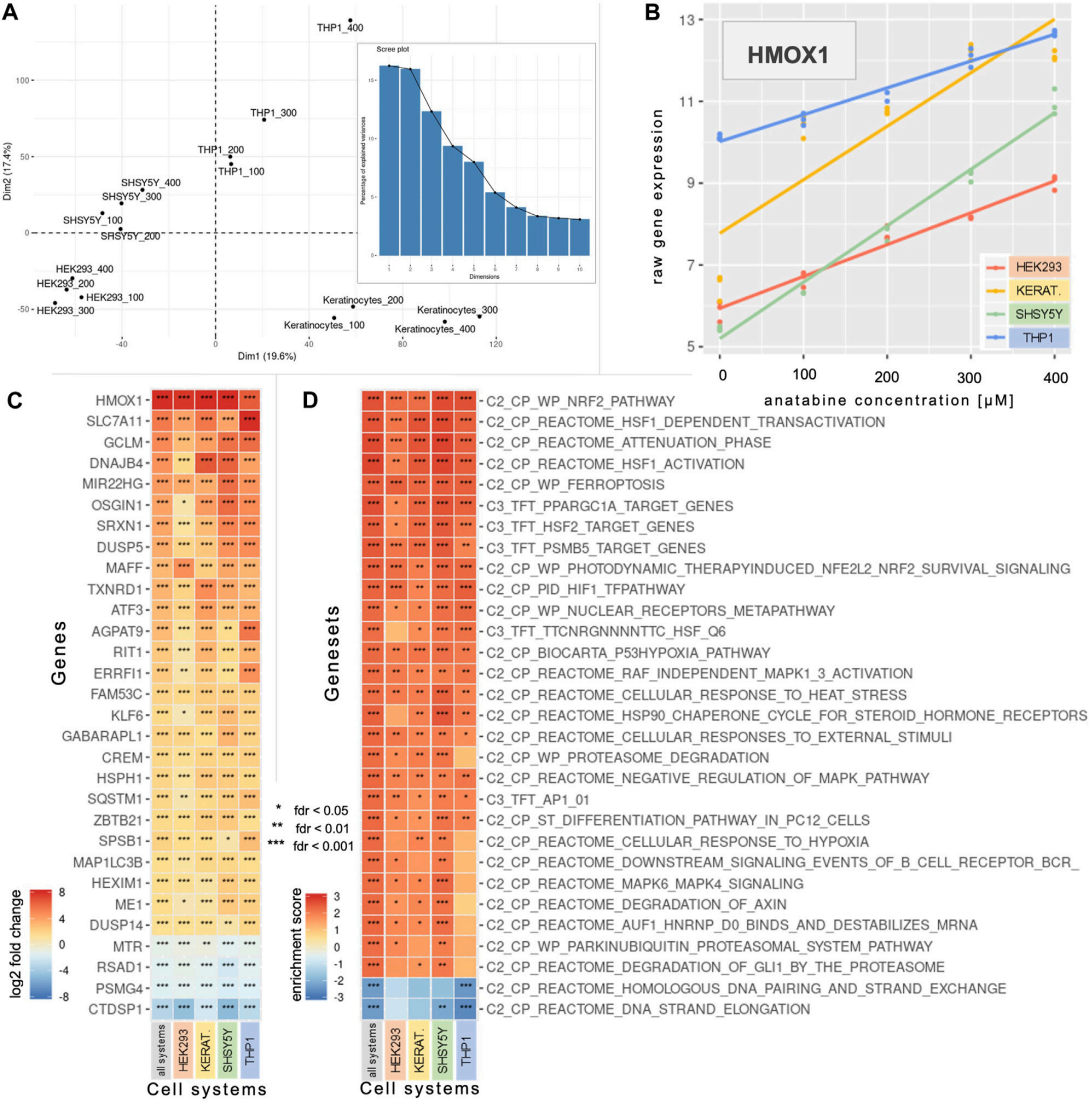
Concentration-dependent effect of anatabine on gene expression in various cell systems. **(A)** The two first dimensions of a PCA across the four anatabine concentrations and the four tested cell systems. The bar plot depicts the percentage of variance explained by each principal component. **(B)** *HMOX1* expression data across the tested anatabine concentrations and cell systems. The slope of each depicted linear model is the value that denotes modifications in the corresponding gene expression with increasing anatabine concentration. **(C)** The 30 most significantly expressed genes based on the “all systems” profile (left column), which captures anatabine’s effect across all tested cell systems and concentrations. The other four columns correspond to the data for each cell system individually. Genes are ranked by median log2 fold change in each row. **(D)** The profiles of panel **C** are subjected to GSEA, and the 30 most significantly enriched gene sets based on the “all systems” profile enrichment are presented here. Gene sets are ranked by median enrichment score in each row. PCA, principal component analysis; GSEA, gene set enrichment analysis; *HMOX1*, heme oxygenase 1.

A principal component analysis enabled us to investigate the sources of variability in the data (Figure 1A). Principal components (PC) 1 and PC2 explained 19.6% and 17.4% of the variability, respectively, highlighting the combined effects of cell type (prominent effect) and anatabine concentrations.

A linear modeling analysis enabled us to identify genes whose expression changed linearly with increasing concentrations of anatabine, for each individual cell system or all cell systems combined (Figures 1B,C). Figure 1B illustrates, as an example, the linear relationship between anatabine concentrations and changes in the expression of the gene heme oxygenase 1 (*HMOX1*) for each of the tested cell systems. The slope of each depicted linear regression line portrays the modification in corresponding gene expression per unit of anatabine concentration. Figure 1C shows the top 30 most significantly expressed genes.

To contextualize the processes and pathways represented by these genes, we conducted a GSEA using various gene set collections, namely “C2-CP” and “C3-TFT”. Figure 1D depicts the 30 most significantly enriched gene sets resulting from the GSEA using the gene list generated from comparing all cell systems together. The most significant positively enriched gene set pertains to the nuclear factor-erythroid 2 p45-related factor 2 (*NRF2*), while the next three are related to the transcription factor heat shock factor 1 (*HSF1*).

### 3.2 Intracellular protein responses to anatabine and their comparison to the gene expression data indicate potential NRF2 pathway activation

To obtain a more systematic view of anatabine’s activity, we investigated intracellular protein perturbations caused by anatabine, on the PMA-differentiated THP-1 cell system, employing DIA mass spectrometry proteomics.

**FIGURE 2:**
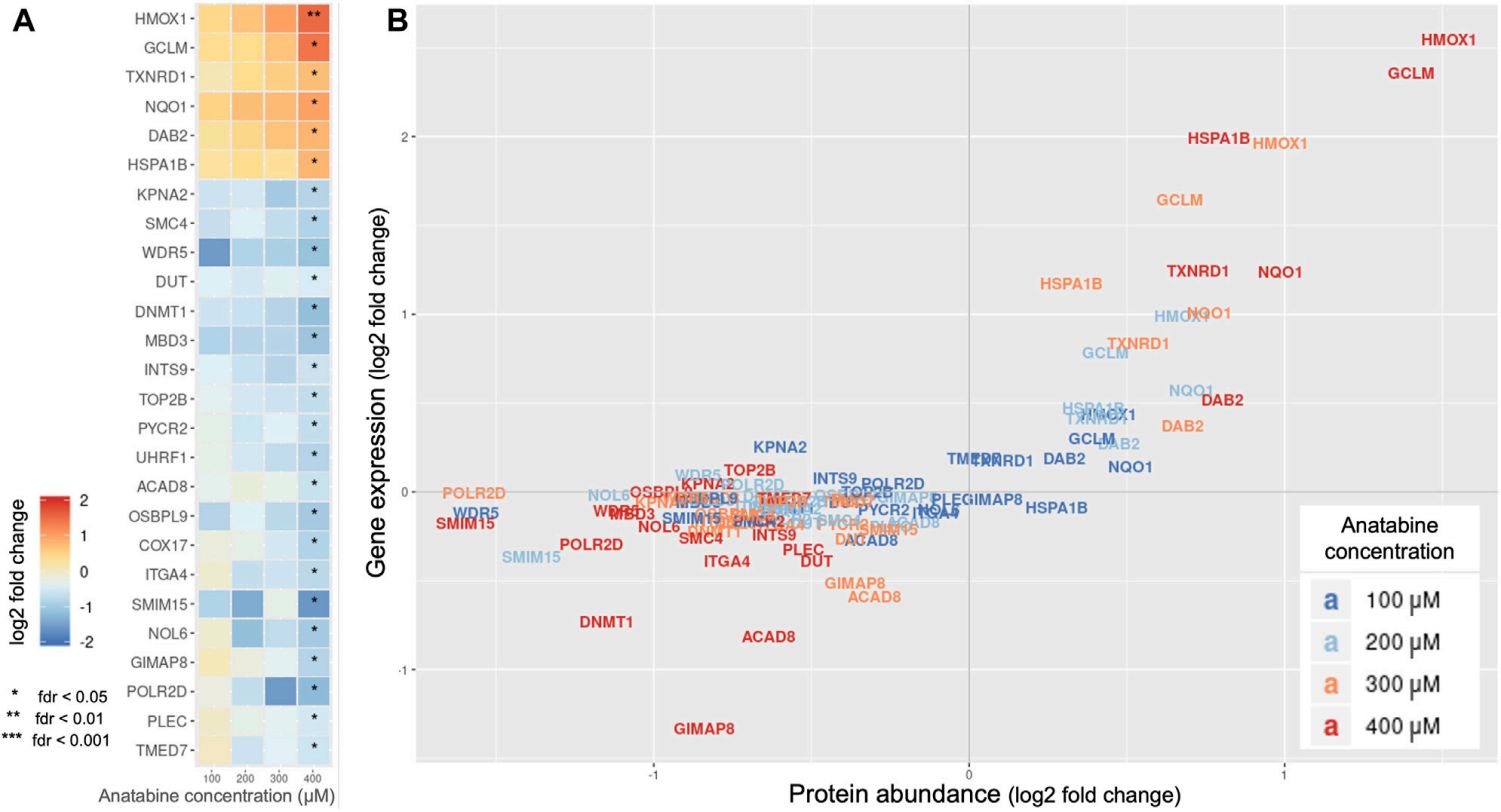
Effect of anatabine on intracellular proteomics. **(A)** Protein abundance upon anatabine treatment of PMA-differentiated THP-1 cells. The proteins shown are the ones significantly expressed (FDR < 0.05). **(B)** Comparison of gene expression and protein abundance upon anatabine treatment. Different anatabine concentrations are color-coded. Only significantly expressed proteins (FDR <0.05) and their corresponding genes are plotted. FDR, false discovery rate.

Figure 2A displays a heatmap of significantly abundant proteins for different concentrations of anatabine. Figure 2B shows the abundance of each protein along with its corresponding gene expression. We observe that the protein abundance of the four most significantly upregulated proteins, HMOX1, glutamate-cysteine ligase modifier subunit (GCLM), thioredoxin reductase 1 (TXNRD1), and NAD(P)H quinone dehydrogenase 1 (NQO1), correlated with the corresponding gene expression changes (Pearson correlation coefficients: 0.97, 0.92, 0.96, and 0.95, respectively).

Based on the observation that these four proteins are NRF2 target genes (Tonelli et al., 2018), we hypothesized that the NRF2 pathway might be involved in anatabine’s mechanism of action.

### 3.3 Anatabine—but no other tobacco alkaloid—causes NRF2 activation

To test our hypothesis, we used an HEK-293 NRF2/ARE luciferase reporter cell line that assesses whether NRF2 translocates to the cell nucleus and binds to its response element upon treatment with the compound. Along with anatabine, we tested the tobacco alkaloids anabasine, cotinine, nornicotine, and nicotine (to investigate the possibility of a shared effect among tobacco alkaloids) and the NRF2-activating compounds sulforaphane and dimethyl fumarate (to serve as the assay’s positive controls).

**FIGURE 3:**
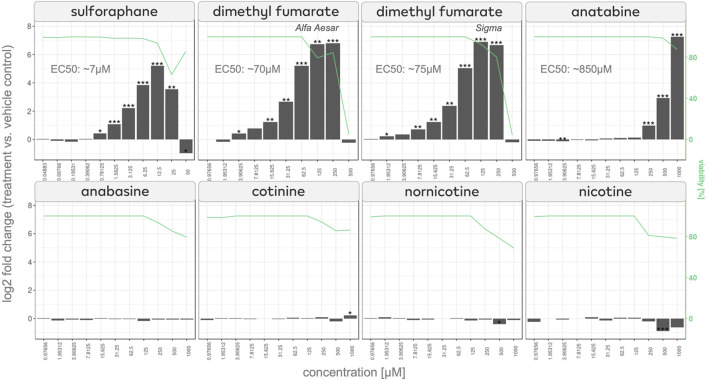
NRF2/ARE luciferase assay of effects of anatabine and other tobacco alkaloids NRF2 activation measured in NRF2 reporter cells after 24 h of stimulation of sulforaphane, dimethyl fumarate, anatabine, anabasine, cotinine, nornicotine, and nicotine. The corresponding cell viability is plotted on the right axis and depicted with a green line.

The results show a concentration-dependent NRF2 activation, which reached statistical significance (*p* < 0.05) at 250 μM of anatabine treatment (Figure 3). Interestingly, anabasine, cotinine, nornicotine, and nicotine did not activate NRF2, demonstrating that NRF2 activation is a unique characteristic of anatabine among the tested tobacco alkaloids.

### 3.4 Anatabine elicits similar response to natural oxidative stress response inducers, regardless of compound structure

To better understand how anatabine activates NRF2 and the molecules that may act as its functional partners, we leveraged the LINCS L1000 dataset that consists of 473,647 differential gene expression signatures (Subramanian et al., 2017), originating from cell systems treated under various conditions with 28,927 different perturbagens, such as chemical compounds, recombinant proteins, or small interfering RNAs. Upon comparing each of those signatures with anatabine’s gene expression profile, we could rank the LINCS perturbagens based on their similarity to anatabine’s profile.

Of the top 15 compounds with gene expression signatures most similar to those of anatabine (Table 1), six have not yet been referenced in the literature, while two other compounds were mentioned in only four studies. The remaining seven compounds are natural compounds that have been shown to induce oxidative stress response. Among them, four compounds have already been identified as NRF2 activators (piperlongumine, withaferin-A, cucurbitacin-I, and parthenolide).

**TABLE 1:** Compounds in the LINCS L1000 dataset with the most similar transcriptomic response to anatabine.

Score	Compound name (LINCS)	PubChem CID	PubMed papers July 2022	Source	Selected mechanism(s) of action
	Anatabine	11388	144	Natural alkaloid extracted from various plants of the Solanaceae family	Induces NRF2 activation
0.49	Piperlongumine	637858	362	Natural alkaloid extracted from long pepper (*Piper longum* L.)	Induces NRF2 activation, through which its differential response in normal and cancer cells is driven Lee et al. (2015)
0.46	NSC-3852	19103	4	Synthetic quinoline	Induces oxidative response, which drives breast cancer cell differentiation Martirosyan et al. (2006); histone deacetylase inhibitor Martirosyan et al. (2004)
0.46	Thiostrepton	16129666	618	Natural antibiotic isolated from various streptomycetes	Induces oxidative response, which drives melanoma cell differentiation Qiao et al. (2012)
0.45	NSC-632839	6477762	4	Synthetic	Apoptosome-independent caspase activation Aleo et al. (2006); nonselective isopeptidase inhibitor Nicholson et al. (2008); UCHL1 inhibitor Yan et al. (2018)
0.45	Withaferin-A	265237	698	Natural lactone extracted from various plants of the Solanaceae family	Induces NRF2 activation, which mediates its antitumor effects Hahm et al. (2021)
0.44	Manumycin-A	73707404	261	Natural antibiotic isolated from *Streptomyces parvulus*	Induces oxidative response and p38 MAPK phosphorylation She et al. (2006); STAT3 inhibitor Dixit et al. (2009); farnesyltransferase inhibitor Giudice et al. (2016)
0.43	CT-200783	73707405	0	Synthetic olefinic compound with functional parent as cinnamic acid	*No reports in the literature*
0.43	GP-42	73707393	0	Synthetic olefinic compound with functional parent as cinnamic acid	*No reports in the literature*
0.42	MD-II-038	51034963	0	Synthetic olefinic compound with functional parent as cinnamic acid	*No reports in the literature*
0.41	BRD-K41172353	673329	0	Synthetic quinoline	*No reports in the literature*
0.41	Cucurbitacin-I	73707401	146	Natural triterpenoid extracted from cucurbitaceous plants	Induces NRF2 activation and STAT/NF-κB inhibition, leading to protection from neuroinflammatory injury Park et al. (2015)
0.41	BRD-K95352812	56643190	0	Synthetic	*No reports in the literature*
0.40	SA-1447005	73707394	0	Synthetic olefinic compound with functional parent as cinnamic acid	*No reports in the literature*
0.40	Radicicol	5311380	511	Natural antibiotic isolated from the fungus *Monosporium bonorden*	Induces oxidative response and heat shock response through HSP90 inhibition and dissociation of HSF1 Ryhanen et al. (2008)
0.40	Parthenolide	7251185	973	Natural lactone extracted from the feverfew plant	Induces NRF2 activation, through which it ameliorates obesity Kim et al. (2019)

**FIGURE 4:**
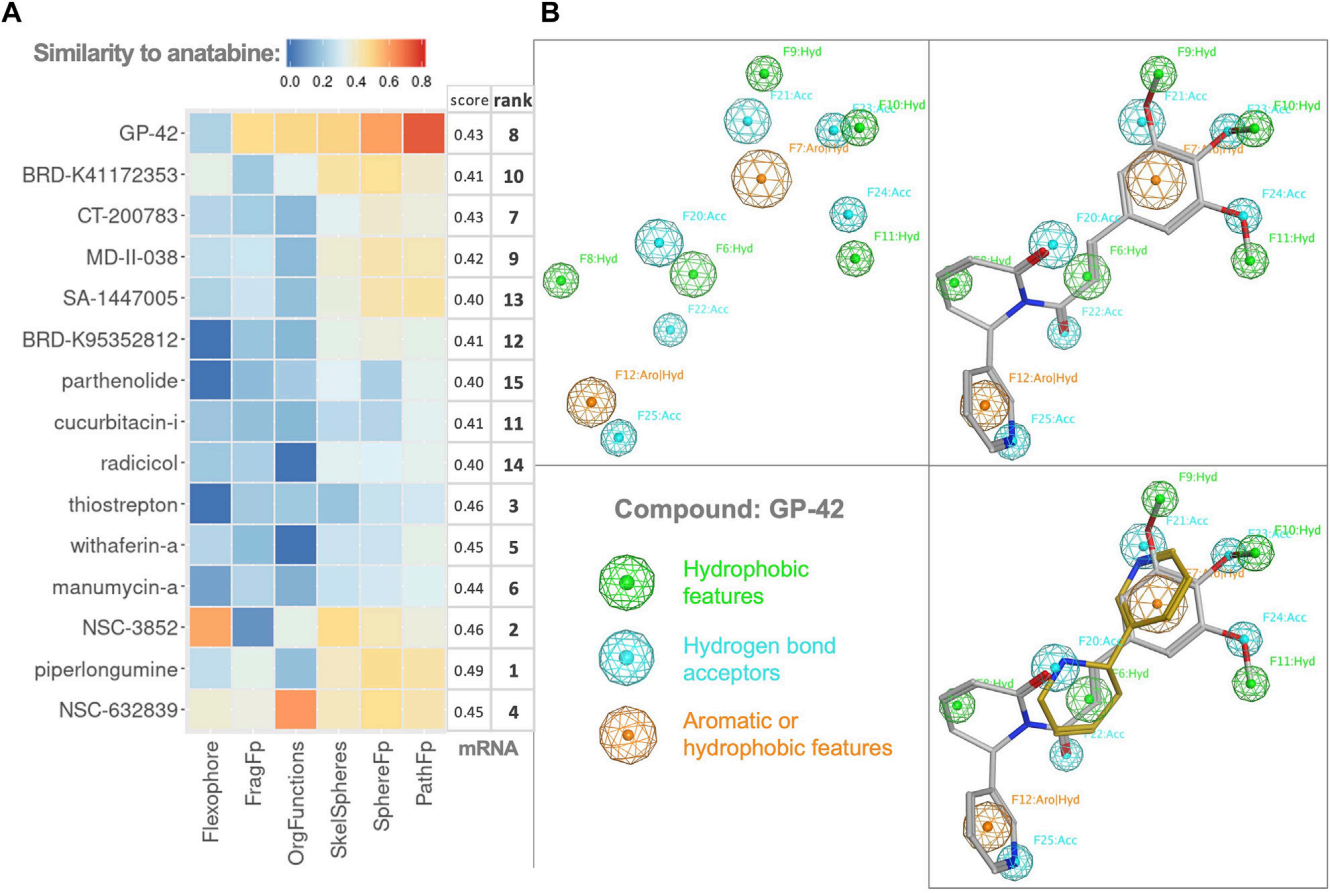
Chemoinformatics. **(A)** Heatmap of Tanimoto distances resulting from the structural similarity comparison of the compounds (on the y-axis) to anatabine. All the tested chemical similarity fingerprints are shown as different columns. The mRNA column corresponds to the similarity in the gene expression score and rank of compounds from Table 1. The heatmap rows and columns are ordered following hierarchical clustering on both axes. **(B)** Pharmacophore model of GP-42. Top left: pharmacophore features of GP-42. Green features represent hydrophobic features, blue features represent hydrogen bond acceptors, and orange features represent features that can be either aromatic or hydrophobic. Top right and bottom right: pharmacophore model fitting for GP-42 and GP-42 together with anatabine, respectively.

We observed common structural elements, such as elements of hydropyridine, piperidine, and quinoline, among the 15 compounds. To examine if the structural elements were responsible for the observed similarities in the gene expression induced by these compounds and anatabine, we employed six different chemical similarity fingerprints and descriptors using the DataWarrior software (Sander et al., 2015). Each compound fingerprint was compared to the corresponding fingerprint of anatabine, and the Tanimoto distances of the structural comparison are presented as a heatmap in Figure 4A.

The correlation between gene expression similarity scores and the chemical similarity fingerprints was examined but not found significant. Compound GP-42 exhibited the highest overall Tanimoto distance when compared with anatabine. Therefore, we visualized the common pharmacophore features of GP-42 and anatabine. A 3D pharmacophore model for GP-42 was generated (Figure 4B). Anatabine was fitted in the pharmacophore model, having one aromatic feature, one hydrophobic feature, and two acceptor features in common with GP-42, demonstrating that anatabine and GP-42 could share a similar mode of binding to some degree.

### 3.5 Anatabine treatment results in the activation of MAPK signaling

We performed network analysis to extract further insights from the transcriptomics data, leveraging the Reactome and Omnipath biological networks (Wu et al., 2010; Turei et al., 2016), the DoRothEA enrichment algorithm (Garcia-Alonso et al., 2019), and the CARNIVAL optimization algorithm (Liu et al., 2019). The resulting networks indicate potential functional partners of anatabine.

**FIGURE 5:**
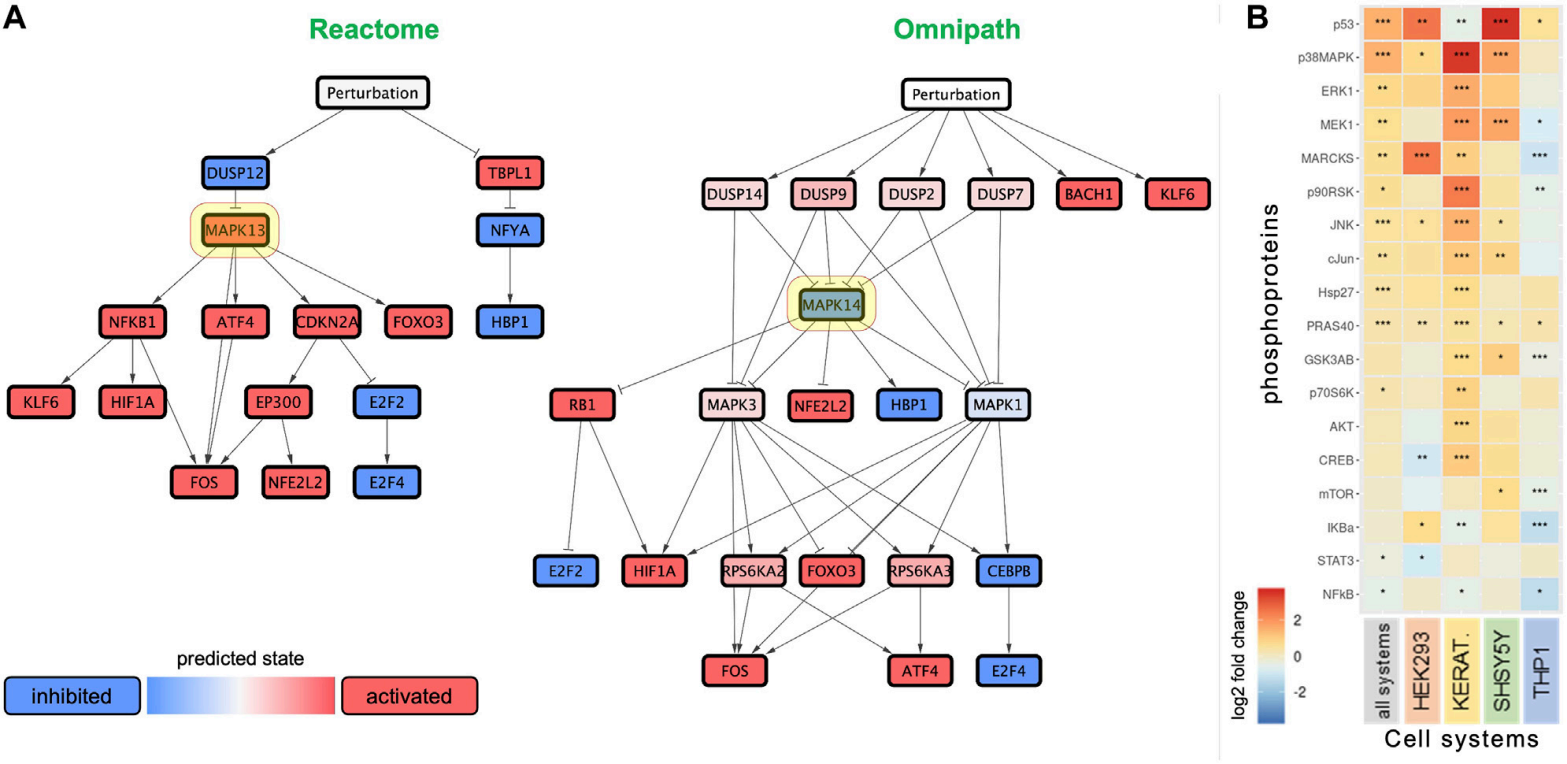
Anatabine treatment results in the activation of MAPK signaling. **(A)** Networks predicting the effect of anatabine, leveraging the biological knowledge networks Reactome and Omnipath. Red and blue nodes depict predicted upregulation and downregulation, respectively. Highlighted in yellow are the network nodes corresponding to p38 MAPK. **(B)** Effect of anatabine on phosphoproteomics across all tested cell systems, treatment times, and concentrations. The other four columns correspond to the data for each cell system separately. Phosphoproteins are ranked by median log2 fold change in each row.

The network analysis revealed p38 MAPK as a central node in the signaling network (Figure 5A). Interestingly, p38 MAPK was predicted to be either positively or negatively regulated depending on the prior knowledge network used in the analysis (Reactome or Omnipath, respectively), even though it was centrally located in both networks. We also observed the presence of dual-specificity phosphatases (DUSP) upstream of the MAPK signaling in both networks. NRF2 was inferred to be activated in both the networks.

To explore the potential involvement of p38 MAPK in facilitating the effects of anatabine, we used multiplexed phosphoproteomic assays. We then applied a linear model approach to capture time- and concentration-dependent phosphoproteomic changes across the four tested cell systems. The phosphoproteomic results after application of the linear model are presented in Figure 5B. The phosphoproteomic data before application of the linear model are detailed in Supplementary Figure S1B.

The phosphoproteomic results, summarized *via* the linear model across time, concentrations, and cell systems tested, revealed a systematic significant increase in the phosphorylation levels of p53, p38 MAPK, ERK1, MEK1, MARCKS, p90RSK, JNK, c-Jun, HSP27, PRAS40, and p70S6K. Differences among cell systems, concentrations, and treatment times are depicted in Figure 5B and Supplementary Figure S1A. Anatabine treatment also caused a significant decrease in the phosphorylation levels in some of the cell systems tested, specifically, a concentration-dependent inhibition of phosphorylated STAT3 and a time-dependent inhibition of phosphorylated NF-κB (Supplementary Figure S1A).

## 4 Discussion

Over the last decade, anatabine has been shown to alleviate inflammation in multiple disease models (Paris et al., 2013b; Ruiz Castro et al., 2020; Xia et al., 2021). However, the molecular mechanisms triggered by anatabine in a non-inflammatory state are yet to be explored. Using a systems biology approach, we investigated the impact of anatabine on various cell systems and identified pathways and molecular networks that are perturbed.

We systematically measured the changes occurring in gene expression and intracellular protein abundance upon anatabine treatment. Abundance of the most significantly upregulated proteins (HMOX1, GCLM, TXNRD1, and NQO1) correlated with changes in their corresponding gene expression, which varied linearly with increasing anatabine concentration. Since these proteins are target genes of the NRF2 transcription factor, and the gene expression enrichment results also pointed to NRF2 pathway activation, we aimed to examine if anatabine induces NRF2 activation.

NRF2 was discovered in 1994 (Moi et al., 1994) as an NF-E2-like basic leucine zipper transcriptional activator. Since then, NRF2 has been highlighted as a master regulator of cytoprotective responses, orchestrating the expression of more than 250 genes (Cuadrado et al., 2018). NRF2 regulates anti-inflammatory gene expression and inhibits the progression of inflammation (Ahmed et al., 2017). However, it is out of the scope of the present study to prove if NRF2 activation is the only mechanism by which anatabine delivers its anti-inflammatory effects, or if there are other mechanisms that are involved and any potential crosstalk between them.

There are several natural product-derived bioactive compounds that are NRF2 activators (Kumar et al., 2014). Our transcriptomics-based comparison of anatabine-regulated genes with the gene expression signatures from the LINCS database, revealed anatabine’s similarity to multiple plant compounds that induce oxidative response, most of which have been reported to activate NRF2 (Table 1). In addition, NRF2 activation has been connected with NF-κB signaling inhibition, a well-known effect of anatabine in inflammation models (Paris et al., 2011). The anti-inflammatory activity of NRF2 was initially thought to rely only on crosstalk with NF-κB, until it was shown that it can directly block LPS-induced transcription of the proinflammatory genes *IL-6* and *IL-1β* in macrophages (Kobayashi et al., 2016). Indeed, NRF2 and NF-κB pathways crosstalk through multiple and complicated mechanisms, including a feedback loop where NF-κB can activate NRF2 and NRF2 activation can attenuate NF-κB signaling (Cuadrado et al., 2018). As an example, sulforaphane, found in broccoli, can attenuate muscle inflammation *via* NRF2-mediated inhibition of the NF-κB signaling pathway (Sun et al., 2015).

Anatabine as well as the alkaloids nicotine, nornicotine, and anabasine can activate α4β2 and α7 nicotinic acetylcholine receptors (nAChR) (Alijevic et al., 2020; Xing et al., 2020). The function of α7nAChR has been linked to the anti-inflammatory mechanism of the phytochemical genistein, which is an NRF2 activator (Guo et al., 2021). However, we found that among the above-mentioned alkaloids, and additionally cotinine, only anatabine is an NRF2 activator, rendering the hypothesis that a nicotinic receptor is actively involved in anatabine-mediated NRF2 activation unlikely. This is particularly intriguing, as the structure of nicotine is the most similar to anatabine than any other compound according to PubChem’s compound similarity search tool (https://pubchem.ncbi.nlm.nih.gov/#query=CID11388%20structure&tab=similarity&fullsearch=true). In fact, nicotine reportedly downregulates NRF2-related activity (Naha et al., 2018; Li et al., 2019). Therefore, the possible mechanisms used by anatabine to cause NRF2 activation are still unclear and must be explored.

Most NRF2 activators, including sulforaphane and dimethyl fumarate that were used in this study, are electrophiles that can modify cysteine residues on Kelch-like ECH-associated protein 1 (KEAP1) (Robledinos-Anton et al., 2019), making the ubiquitylation of NRF2 for proteasomal degradation impossible. Interestingly, anatabine is a nucleophilic compound; therefore, it most probably is unable to interact with KEAP1 in the way other electrophilic compounds do.

Natural compounds can activate NRF2 through different mechanisms, involving diverse molecular processes that we have just started to understand. For example, we have found that the natural lactone withaferin-A, extracted from various plants of the Solanaceae family, has a transcriptomic profile similar to that of anatabine. Withaferin-A increases NRF2 levels through the PTEN/PI3K/AKT/GSK3β axis (Palliyaguru et al., 2016; Liu et al., 2021).

Our phosphoproteomic studies revealed an increase in the phosphorylation of a broad range of MAPK signaling members, including p38 MAPK, ERK1, MEK1, and c-Jun N-terminal kinase (JNK), across the cell systems tested. Additionally, we report a significant increase in the phosphorylation of c-Jun, which is the main cellular substrate activated by JNK-mediated phosphorylation (Yarza et al., 2015). All well-characterized categories of MAPKs have been linked to NRF2 regulation but with contradictory results reported (Sun et al., 2009). Specifically, p38 MAPK has been reported to regulate NRF2 both positively and negatively among different groups (Liu et al., 2021). Interestingly, our network analysis supports this finding as p38 MAPK was predicted to be either inhibited or activated in different models; however, it was always a central node of anatabine’s predicted network of perturbations. Piperlongumine, a natural alkaloid extracted from long pepper (*Piper longum* L.), was identified as the most similar compound to anatabine, based on analysis of the transcriptomic profiles of the 28,927 compounds. JNK inhibition blocks the piperlongumine-induced NRF2 translocation (Mohammad et al., 2019), suggesting the involvement of MAPK signaling. Niacin, the compound from which anatabine derives both its pyridine rings (Kaminski et al., 2020), activates NRF2 and the p38 MAPK signaling pathway (Wu et al., 2012).

In addition, we observed an increased phosphorylation of p53. Reportedly, a non-esterified fatty acid that increased the phosphorylation of ERK1 and p38 MAPK, also upregulated and caused the translocation of p53 and NRF2, suggesting that its mechanism of action was mediated by the NRF2/p53 signaling pathway (Wang et al., 2020).

Several NRF2 activators have been reported to trigger many of the above mechanisms at the same time, and it has been suggested that these pleiotropic effects are one of the reasons that drug development of NRF2 activators is moving slowly (Robledinos-Anton et al., 2019). Beyond NRF2, our GSEA also revealed transcriptional activation of HSF1, which may be of interest for further experimental investigations, given that the two transcription factors, NRF2 and HSF1, engage in crosstalk for cytoprotection by promoting the reduced state (Dayalan Naidu et al., 2015).

It also remains to be explored how anatabine activates MAPK signaling. Our network analysis pointed to the inhibition of several DUSPs, which are negative regulators of MAPK signaling, that dephosphorylate p38, JNK, and ERK in different settings (Ramkissoon et al., 2019). Further experiments utilizing inhibitors would be necessary to confirm that anatabine-mediated NRF2 activation depends on any of these kinases, which is out of the scope of the current study.

Regarding chemoinformatics, our analysis of compounds with similar transcriptomic response to anatabine revealed some common structural elements; however, the chemical similarity fingerprints examined were not significantly correlated to the gene expression similarity scores, potentially showing that certain structural elements are not necessary for the observed transcriptomic response.

Given the critical role of NRF2 in chronic diseases, there has been an increasing interest from the pharmaceutical industry in the discovery and clinical development of small molecule NRF2 inducers (Cuadrado et al., 2019). A plethora of NRF2 activators have been identified, and some of them are under clinical development, especially those for chronic diseases characterized by low-grade oxidative stress and inflammation (Robledinos-Anton et al., 2019). More established NRF2 activators have already been rigorously researched. Sulforaphane has been used in at least 32 clinical studies to date, addressing chronic diseases such as cancer, asthma, chronic kidney disease, and cystic fibrosis (Cuadrado et al., 2018). The use of dimethyl fumarate in patients with multiple sclerosis was propelled by positive results obtained in a multiple sclerosis mouse model of EAE, much like it has already been observed for anatabine (Paris et al., 2013b). To date, dimethyl fumarate is the only drug approved by the Food and Drug Administration and European Medicines Agency and registered as an NRF2 activator. Going one step further, analogs of NRF2 activators are being developed for optimizing efficacy and better manage pleiotropic or off-target effects, such as in the case of piperlongumine (Ji et al., 2021).

While there are certain areas to be explored in deciphering the anti-inflammatory mechanisms of action of anatabine and other NRF2 activators, we believe that anatabine constitutes an interesting molecule for its therapeutic potential in NRF2-related diseases.

## Data Availability

The original contributions presented in the study are included in the Supplementary Material, further inquiries can be directed to the corresponding author.
