# Supplementary figures and images for: Systems biology reveals anatabine to be an NRF2 activator
Source: Front Pharmacol. 2022 Nov 16;13:1011184. doi: 10.3389/fphar.2022.1011184 (PMC9708905; doi:10.3389/fphar.2022.1011184)

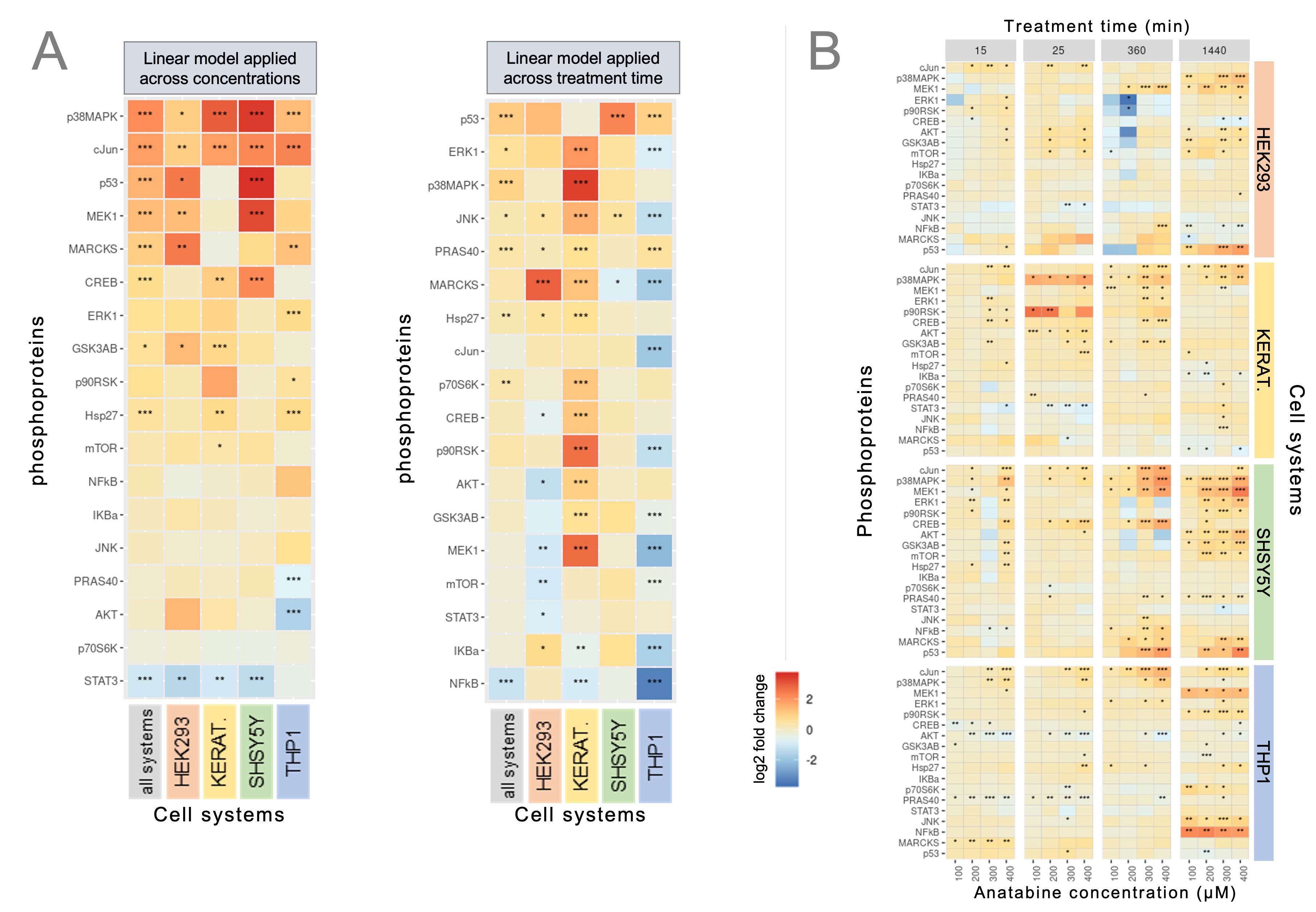

Supplement: Supplementary file 1 [file Image1.JPEG]

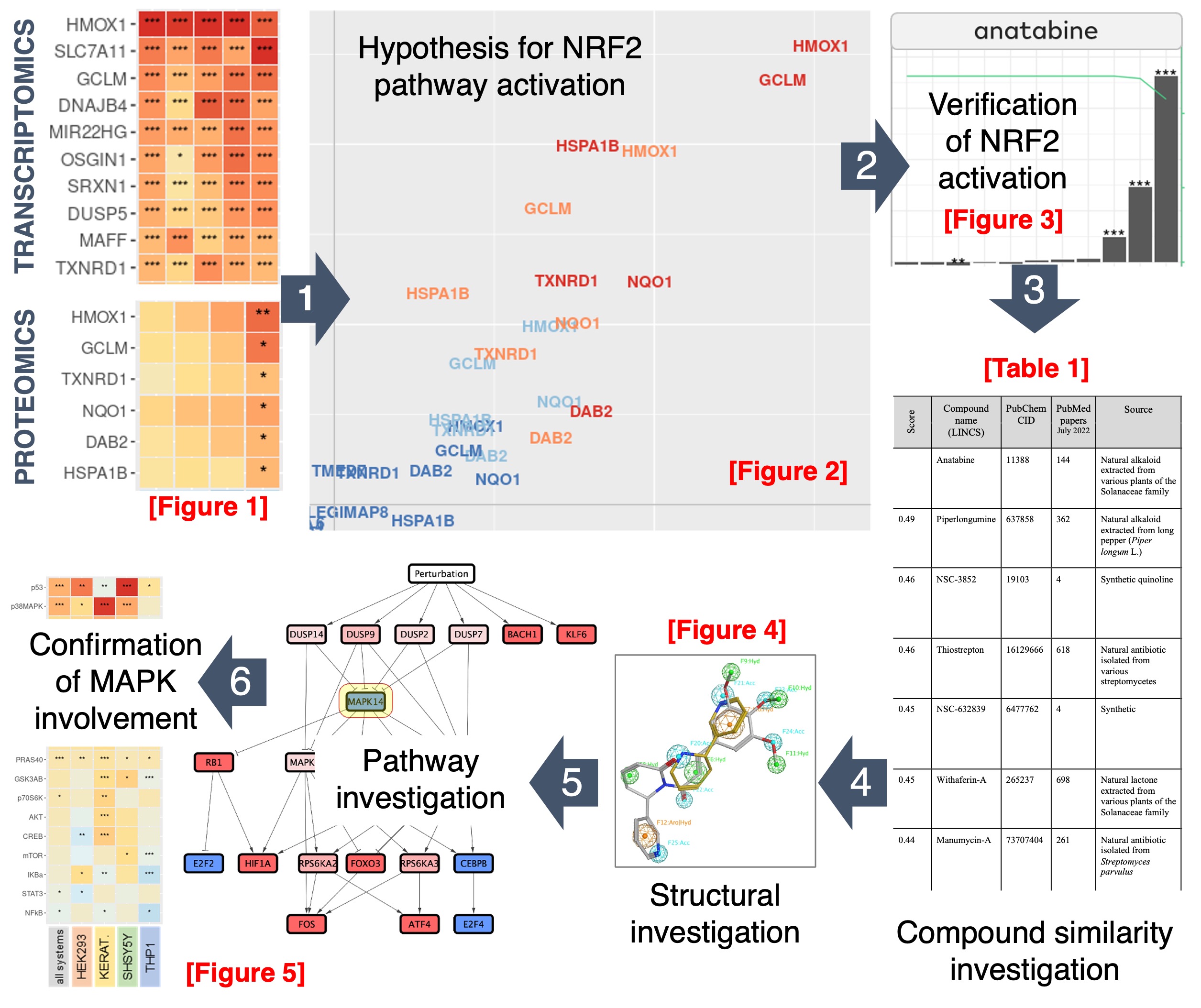

Supplement: Supplementary file 2 [file Image2.JPEG]
